# Factors affecting the health-promoting behavior of thyroid cancer survivors: comparison by stage of cancer survivorship

**DOI:** 10.1007/s00520-022-06799-9

**Published:** 2022-01-08

**Authors:** Kyung Ah Park, Sanghee Kim, Eui Geum Oh, Heejung Kim, Hang-Seok Chang, Soo Hyun Kim

**Affiliations:** 1 Thyroid Cancer Center, Gangnam Severance Hospital, Yonsei University Health System, Seoul, Korea; 2 Division of Nursing, Gangnam Severance Hospital, Yonsei University Health System, Seoul, Korea; 3 College of Nursing, Yonsei University, Seoul, Korea; 4 Mo-Im Kim Nursing Research Institute, Yonsei University, Seoul, Korea; 5 Department of Surgery, Yonsei University College of Medicine, Seoul, Korea; 6 Institute of Refractory Thyroid Cancer, Yonsei University College of Medicine, Seoul, Korea; 7 Department of Nursing, Inha University, Incheon, Korea

**Keywords:** Health-promoting behavior, Thyroid cancer survivors, Survival stage, Social support

## Abstract

**Purpose:**

The purpose of this study was to identify differences in factors affecting health-promoting behaviors according to the survival stage of thyroid cancer survivors.

**Methods:**

This descriptive cross-sectional study analyzed data from 354 thyroid cancer survivors after diagnosis. The survivors were divided into three stages: (1) the acute stage (< 2 years after diagnosis), (2) extended stage (2–5 years after diagnosis), and (3) permanent stage (≥ 5 years after diagnosis). To measure health-promoting behavior, the revised Korean version of the Health Promoting Lifestyle Profile questionnaires was used. The factors affecting the health-promoting behavior included social support, self-efficacy, fear of recurrence, and symptoms. Multiple regression analysis was used to analyze factors affecting the health-promoting behavior according to survival stage.

**Result:**

The factors affecting the health-promoting behavior of thyroid cancer survivors differed by survival stage. In the acute stage, the factors of health-promoting behavior were self-efficacy (*t* = 4.76, *p* < .001) and social support (*t* = 3.54, *p* < .001). In the extended stage, symptoms (*t* =  − 3.65, *p* < .001), social support (*t* = 2.61, *p* = .011), fear of recurrence (*t* = 2.18, *p* = .032), and receipt of radioiodine treatment (*t* =  − 2.18, *p* = .032) were found to be significant variables that affected health-promoting behaviors. In the permanent stage, social support (*t* = 2.79, *p* = .007), receipt of radioiodine treatment (*t* =  − 3.21, *p* = .002), and age (*t* =  − 2.77, *p* = .007) were significant variables that affected health-promoting behaviors.

**Conclusion:**

The experience of thyroid cancer survivors varies as they progress through the survival stages; thus, health-promotion interventions should be tailored to each survival stage.

## Introduction

The incidence of thyroid cancer is increasing worldwide, and the 5-year survival rate for patients is very high (98–100%) [1–3]. Thyroid cancer is the most common cancer in young adults, with the highest rate of diagnosis in Korea (28.3%), among young adults in their 40 s [3, 4]. Because thyroid cancer has better prognosis, a much higher survival rate, and earlier onset than other adult-cancers, long-term healthcare for thyroid cancer survivors is very important [1–5].

Patients with cancer continue to experience symptoms after treatment and need healthcare interventions applied to their daily lives [6]. Patients with thyroid cancer have similar or lower quality of lives compared to other cancer patients, and they have been shown to experience various side effects after treatment [7, 8]. In a previous study, thyroid cancer survivors experienced more anxiety, depression, fatigue, and sleep disturbances than other cancer survivors [9].

Proper health promotion among cancer patients plays a key role in improving the quality of life related to health [10]. Therefore, it is necessary to be aware of the factors related to patients’ willingness to engage in health-promoting behaviors. Health promotion is the process of improving one’s own health and can be described using six areas: health responsibility, self-realization, physical activity, nutrition habits, interpersonal relations, and stress management [11, 12]. In addition, personal characteristics, self-efficacy, social support, and perceived threats are known to affect the performance of health-promoting behaviors [11, 13]. In a previous study in cancer survivors, health behaviors differed according to the time of diagnosis, physical symptoms, and fear of recurrence [14, 15]. Patients with thyroid cancer are predicted to have different health behaviors depending on their individual disease-related characteristics, but few studies have reported the health-promoting behaviors of thyroid cancer patients.

Because thyroid cancer survivors need long-term follow-up, for management of comorbidities, and prevention of secondary cancer, they need to engage in health-promoting behaviors throughout their lives [16, 17]. The needs of cancer survivors for healthcare and services are classified into the acute stage (< 2 years after cancer diagnosis), extended stage (2–5 years after cancer diagnosis), and permanent stage (≥ 5 years after cancer diagnosis) [18]. Medical staff need to understand the emotional and physical problems that occur as cancer survivors proceed through treatment.

A diagnosis of cancer can change patients’ health behaviors. Patients diagnosed with cancer within a month showed improved physical activity and dietary changes [19]. In the case of patients with gynecological cancers, health-promoting behaviors were significantly higher in the acute stage than in the extended stage [20]. In addition, long-term cancer survivors (≥ 5 years after cancer diagnosis) reported no differences from the general population, in screening behavior for secondary diseases, or physical activity. Their interest in the physical aspects of health was reduced compared to the psychological and social aspects [21, 22]. In summary, cancer survivors showed good health-promoting behaviors in the acute stage, which seemed to decline over time.

In patients with long-term survival for thyroid cancer, the factors affecting health-promoting behaviors may change depending on the stage of survival. Few studies have examined the differences in survival stages of patients with thyroid cancer. Therefore, this study identified differences among thyroid cancer survivors according to survival stage, along with the factors that influence health-promoting behaviors.

## Materials and methods

### Setting and participants

This was a cross-sectional and descriptive correlation study. This study analyzed the level of social support, self-efficacy, fear of recurrence, symptoms, and health-promoting behaviors of patients with thyroid cancer, along with factors that affect health-promoting behaviors at each stage of survival. The subjects of this study were adults (≥ 18 years) who had undergone surgery to treat thyroid cancer. Those who were diagnosed with a cancer other than thyroid cancer were excluded. This study collected data from patients visiting an outpatient clinic or a thyroid cancer online community from December 30, 2019, to January 31, 2020.

First, the study was explained to subjects who visited the outpatient clinic and met the selection criteria. If they agreed to participate in the study, they completed the paper questionnaire. Questionnaires were administered to 45 patients, and two who refused to participate were excluded. Second, we received an online questionnaire through the thyroid cancer patient community (https://cafe.naver.com/thyroidfamily) opened on the “Naver” portal site. The community has about 30,000 thyroid patients and provides information related to thyroid cancer, and anyone can join without a subscription fee. After posting a research notice on an online community bulletin board, questionnaires were received from those who voluntarily agreed to participate in the study through “SurveyMonkey” (https://en.surveymonkey.com/). There was no compensation for patient participation in this study. Questionnaire responses were collected from 43 outpatients, and 317 patients who responded online. Data analysis included 354 patients, excluding 6 patients diagnosed with secondary cancers other than thyroid cancer. By survival stage, 147 respondents were in the acute stage, 112 in the extended stage, and 95 in the permanent stage.

This study was approved by the hospital’s institutional review board. The participants were given the researcher’s email address and telephone number. To protect personal information, all data were processed anonymously, and access to people other than the researcher was restricted.

### Survey instrument

The questionnaire used in this study contained 103 items on individual characteristics, health-promoting behaviors, social support, self-efficacy, fear of recurrence, and symptoms.

Information on individual characteristics collected include age, sex, marital status, education, monthly family income, employment status, frequency accessing online community, cancer type, extent of surgery, cancer recurrence, and number of I-131(radioactive iodine) therapies received. The treatment of thyroid cancer is generally thyroidectomy, and if there is a risk of recurrence, additional I-131(radioactive iodine) therapy is performed [23].

To measure health-promoting behavior, the Health Promoting Lifestyle Profile (HPLP) developed by Pender was translated by Oh and Hong and edited to make it applicable to survivors of thyroid cancer [11, 12]. The HPLP consists of six sub-areas: self-realization, health responsibility, exercise, nutrition, interpersonal relationships, rest, and stress management. In this study, the original questionnaire was modified and supplemented to a total of 38 questions: 7 questions about self-actualization, 11 about health responsibility, 4 about exercise, 5 about nutrition, 5 about interpersonal relationships, and 6 about relaxation and stress management. All questions were answered using a 4-point Likert scale, in which a higher score indicated higher participation in health-promoting behaviors. In this study, Cronbach’s *α* = 0.90, which indicates the reliability of the HPLP in measuring health-promoting behaviors.

Social support was measured using the Multidimensional Scale of Perceived Social Support (MSPSS) developed by Zimet et al. and translated by Shin and Lee [24, 25]. The MSPSS is a 12-item scale with three subscales: family, friends, and significant others. The questions were answered using a 5-point Likert scale, in which a higher score indicated better social support. At the time of tool development, the MSPSS had a Cronbach’s *α* of 0.83, and in this study, Cronbach’s *α* = 0.94.

The Cancer Survivors’ Self-Efficacy Scale (CSSES) was developed by Foster et al. [26]. This study used the Korean version (CSSES-K) developed by Kim et al. [27]. It contains 10 questions, each of which is given 1 to 10 points on a Likert scale, with higher item scores indicating higher self-efficacy. In this study, Cronbach’s *α* for the CSSES-K was 0.91.

The Fear of Progression Questionnaire (FOP-Q) developed by Herschbach et al. was edited by Mehnert et al. to reduce the number of questions for cancer patients (FOP-Q-12) [28, 29]. This study used the FOP-Q-12 in Korean, developed by Shim et al. [30]. Each of the 12 questions is given 1 to 5 points on a Likert scale, with higher scores indicating a greater fear of cancer recurrence. In this study, the Cronbach’s *α* was 0.90.

For the symptoms, this study used the Quality of Life Tool for Korean thyroid cancer survivors (KT-QoL) based on the original work on the thyroid cancer–specific, City of Hope-QOL Scale [31, 32]. The 14 original KT-QoL questions confirmed the physical aspects of the symptoms of patients with thyroid cancer: fatigue, skin/hair changes, sleep, cold/heat intolerance, pain, weight gain, voice changes, dysfunction of motor skills, feeling of something stuck in the neck, fluid retention, infertility, loss of appetite, constipation, and difficulty swallowing. Two questions were added in this study: numbness of the hands and feet, and diarrhea. A sum of the 16 items was used for the symptom score. Each item is given 0 to 10 points on a Likert scale, with higher item scores indicating more severe symptoms. Cronbach’s *α* for the 16 questions used in this study was 0.89.

### Data analysis

The data collected were analyzed using IBM Statistical Package for Social Sciences (SPSS) version 25.0. Differences according to survival stage were analyzed using the *χ*^2^ test. Differences in the performance of health-promoting behavior, personal characteristics, social support, self-efficacy, fear of recurrence, and symptoms by survival stage were analyzed using one-way analysis of variance (ANOVA), and post-analysis was conducted using the Tukey HSD test. Correlations between the use of health-promoting behaviors and social support, self-efficacy, fear of recurrence, and symptoms were analyzed using Pearson’s correlation coefficients. Multiple regression analyses were performed to determine the factors that affect participation in health-promoting behaviors.

## Results

### Characteristics of subjects by survival stage

Table 1 shows the characteristics of the study participants. The 40 s age group had the most subjects (42.2%), and 91.2% of them were women. Significant differences by survival stage were found in age, online community access, surgical scope, recurrence experience, and the number of radioactive iodine treatments received. In the acute stage, the highest percentage was from the number of patients accessing the online community daily (68%). In the permanent stage, 20.0% of people were in their 60 s or older, a higher age group than in other survival stages; 38.9% of respondents had a lateral resection and re-operation, and 15.8% experienced a recurrence. In the acute stage, 4.1% of patients received radioactive iodine treatment more than twice, whereas in the extended survival it was 21.4%, and 31.6% in the permanent stage.

**Table 1:** Characteristics of thyroid cancer survivors by survival stage

Characteristics	Categories	Total (*n* = 354)	Acute survival (*n* = 147)	Extended survival (*n* = 112)	Permanent survival (*n* = 95)	*χ*^2^	*p*
		*n* (%)	*n* (%)	*n* (%)	*n* (%)		
Age (years)							
	< 40	96 (27.2%)	46 (31.5%)	37 (33.0%)	13 (13.7%)	28.82	< .001
	40–49	149 (42.2%)	60 (41.1%)	49 (43.8%)	40 (42.1%)		
	50–59	77 (21.8%)	34 (23.3%)	20 (17.9%)	23 (24.2%)		
	≥ 60	31 (8.8%)	6 (4.1%)	6 (5.4%)	19 (20.0%)		
Gender							
	Men	31 (8.8%)	13 (8.8%)	10 (8.9%)	8(8.4%)	0.02	0.991
	Women	323 (91.2%)	134 (91.2%)	102 (91.1%)	87(91.6%)		
Marital status							
	Married	291 (82.2%)	117 (79.6%)	94 (83.9%)	80(84.2%)	1.18	0.556
	Single	63 (17.8%)	30 (20.4%)	18 (16.1%)	15 (15.8%)		
Education							
	≤ High school	78 (22.0%)	27 (18.4%)	26 (23.2%)	25(26.3%)	2.26	0.324
	≥ College	276 (78.0%)	120 (81.6%)	86 (76.8%)	70(73.7%)		
Monthly income							
(10,000 Korean won)^1^	< 200	19 (5.4%)	8 (5.5%)	6 (5.4%)	5 (5.3%)	3.36	0.763
	200 to < 400	100 (28.4%)	44 (30.1%)	26 (23.2%)	30 (31.9%)		
	400 to < 600	118 (33.5%)	51 (34.9%)	40 (35.7%)	27 (28.7%)		
	≥ 600	115 (32.7%)	43 (29.5%)	40 (35.7%)	32 (34.0%)		
Employment status							
	Employed	205 (57.9%)	85 (57.8%)	71 (63.4%)	49(51.6%)	2.94	0.229
	Not employed	149 (42.1%)	62 (42.2%)	41 (36.6%)	46(48.4%)		
Frequency accessing online community							
	Every day	191 (54.0%)	100 (68.0%)	46 (41.1%)	45 (47.4%)	29.42	< .001
	2–3 times a week	80 (22.6%)	29 (19.7%)	31 (27.7%)	20 (21.1%)		
	2–3 times a month	57 (16.1%)	11 (7.5%)	28 (25.0%)	18 (18.9%)		
	Rarely accessed	26 (7.3%)	7 (4.8%)	7 (6.3%)	12 (12.6%)		
Cancer type							
	Papillary	338 (95.5%)	143 (97.3%)	104 (92.9%)	91(95.8%)	2.91	0.234
	Other^2^	16 (4.5%)	4 (2.7%)	8 (7.1%)	4 (4.2%)		
Extent of surgery							
	Less-than-total^3^	150 (42.5%)	76 (52.1%)	47 (42.0%)	27(28.4%)	17.53	0.002
	Total thyroidectomy	112 (31.7%)	43 (29.5%)	38 (33.9%)	31(32.6%)		
	Other^4^	91 (25.8%)	27 (18.5%)	27 (24.1%)	37(38.9%)		
Cancer recurrence							
	Yes	28 (7.9%)	6 (4.1%)	7 (6.3%)	15(15.8%)	11.48	0.003
	No	326 (92.1%)	141 (95.9%)	105 (93.8%)	80(84.2%)		
Number of I¹³¹ therapies^5^							
	None	181 (51.1%)	96 (65.3%)	56 (50.0%)	29(30.5%)	42.91	< .001
	1 time	113 (31.9%)	45 (30.6%)	32 (28.6%)	36(37.9%)		
	≥ 2 times	60 (16.9%)	6 (4.1%)	24 (21.4%)	30 (31.6%)		

^1^Incomes are shown in 10,000 South Korean won (KRW) units

^2^Other includes follicular cancer, Hurthle cell cancer, medullary cancer, poorly differentiated cancer, and anaplastic cancer

^3^Less-than-total includes less-than-total thyroidectomy and hemithyroidectomy

^4^Other includes total thyroidectomy along with lateral neck dissection and re-operation

^5^I^131^ therapies mean radioactive iodine therapies

### The sub-areas of health-promoting behavior

The total score for health-promoting behavior was 101.32 ± 15.09, and the average item score was 2.67 ± 0.40. In the sub-areas of health-promoting behavior, the score for health responsibility was highest at 2.83 ± 0.39 points, and the score for exercise was the lowest at 2.36 ± 0.77 points (Table 2).

**Table 2:** Levels of health-promoting behavior, social support, self-efficacy, fear of recurrence, and symptoms

Variables	Mean ± SD	Range	Number of items	Item mean ± SD	Item range
Health-promoting behavior	101.32 ± 15.09	63–144	38	2.67 ± 0.40	1–4
Self-realization	18.68 ± 3.76	9–28	7	2.67 ± 0.54	1–4
Health responsibility	31.23 ± 4.25	19–44	11	2.83 ± 0.39	1–4
Physical activity	9.42 ± 3.08	4–16	4	2.36 ± 0.77	1–4
Nutrition	13.21 ± 2.87	6–20	5	2.64 ± 0.57	1–4
Interpersonal relations	14.01 ± 2.86	5–20	5	2.80 ± 0.57	1–4
Stress management	14.77 ± 3.48	6–23	6	2.46 ± 0.58	1–4
Social support	45.61 ± 9.91	16–60	12	3.80 ± 0.83	1–5
Family	15.90 ± 3.76	4–20	4	3.96 ± 0.94	1–5
Friends	14.15 ± 3.92	4–20	4	3.54 ± 0.98	1–5
Significant others	15.56 ± 3.61	4–20	4	3.89 ± 0.90	1–5
Self-efficacy	64.20 ± 17.23	10–100	10	6.42 ± 1.72	1–10
Fear of recurrence	40.79 ± 10.78	12–60	12	3.40 ± 0.90	1–5
Symptoms	55.80 ± 29.11	0–134	16	3.49 ± 1.82	0–10

*SD* standard deviation

### Differences in health-promoting behavior, social support, self-efficacy, fear of recurrence, and symptoms by survival stage

The scores with significant differences by survival stage were social support and fear of recurrence. The social support score was 46.69 ± 9.15 points in the extended stage, which was significantly higher than the 43.59 ± 10.26 points in the permanent stage; fear of recurrence was 42.52 ± 10.27 points in the acute stage, significantly higher than the 38.63 ± 11.73 points in the permanent stage (Table 3).

**Table 3:** Differences in health-promoting behavior, social support, self-efficacy, fear of recurrence, and symptoms by survival stage

Variables	Total (*n* = 354)	Acute survival ^a^ (*n* = 147)	Extended survival ^b^ (*n* = 112)	Permanent survival ^c^ (*n* = 95)	*F*(*p*)
	Mean ± SD	Mean ± SD	Mean ± SD	Mean ± SD	
Health-promoting behavior	101.32 ± 15.09	100.75 ± 15.15	102.11 ± 13.96	101.26 ± 16.33	0.26(.773)
Self-realization	18.68 ± 3.76	18.24 ± 3.94	19.28 ± 3.38	18.66 ± 3.83	2.45(.088)
Health responsibility	31.23 ± 4.25	31.36 ± 4.34	31.14 ± 4.28	31.15 ± 4.13	0.11(.896)
Physical activity	9.42 ± 3.08	9.37 ± 3.01	9.23 ± 2.86	9.69 ± 3.43	0.60(.549)
Nutrition	13.21 ± 2.87	13.14 ± 2.73	13.26 ± 3.11	13.25 ± 2.82	0.07(.936)
Interpersonal relations	14.01 ± 2.86	13.87 ± 3.04	14.21 ± 2.47	13.98 ± 3.02	0.44(.645)
Stress management	14.77 ± 3.48	14.76 ± 3.37	14.99 ± 3.50	14.53 ± 3.64	0.46(.634)
Social support	45.61 ± 9.91	45.89 ± 10.08	46.96 ± 9.15	43.59 ± 10.26	3.10(.046)^b>c^
Family	15.90 ± 3.76	16.01 ± 3.87	16.34 ± 3.47	15.22 ± 3.87	2.40(.092)
Friends	14.15 ± 3.92	14.14 ± 4.08	14.43 ± 3.75	13.83 ± 3.88	0.60(.552)
Significant others	15.56 ± 3.61	15.74 ± 3.53	16.19 ± 3.37	14.54 ± 3.81	5.86(.003)^a,b>c^
Self-efficacy	64.20 ± 17.23	64.31 ± 17.81	64.53 ± 13.61	63.64 ± 20.08	0.72(.930)
Fear of recurrence	40.79 ± 10.78	42.52 ± 10.27	40.35 ± 10.30	38.63 ± 11.73	3.95(.020)^a>c^
Symptoms	55.80 ± 29.11	56.08 ± 28.59	53.20 ± 28.53	58.42 ± 30.61	0.84(.433)
Fatigue	5.56 ± 2.69	5.71 ± 2.55	5.75 ± 2.59	5.08 ± 3.00	2.20(.134)
Skin/hair change	4.39 ± 3.09	4.51 ± 3.01	4.43 ± 3.13	4.17 ± 3.06	0.36(.696)
Sleep	4.35 ± 3.11	4.45 ± 3.16	4.07 ± 2.93	4.52 ± 3.26	0.66(.517)
Cold/heat sensitive	4.34 ± 3.23	4.13 ± 3.21	4.54 ± 3.17	4.42 ± 3.34	0.57(.568)
Pain	4.22 ± 2.94	4.39 ± 3.01	4.02 ± 2.80	4.19 ± 3.01	0.51(.602)
Weight gain	3.81 ± 3.38	3.61 ± 3.53	3.64 ± 3.34	4.31 ± 3.16	1.43(.240)
Voice change	3.61 ± 3.21	3.79 ± 3.25	2.84 ± 3.87	4.25 ± 3.67	5.50(.004)^b^^<^^a, c^
Motor skills	3.61 ± 2.87	3.96 ± 3.03	3.01 ± 2.52	3.79 ± 2.93	3.77(.024)^a>b^
Something stuck in the neck	3.49 ± 3.17	3.59 ± 3.32	3.16 ± 2.84	3.73 ± 3.31	0.94(.392)
Fluid retention	3.30 ± 3.06	3.22 ± 3.10	3.25 ± 2.96	3.47 ± 3.14	0.21(.814)
Fertility	3.18 ± 3.18	3.18 ± 3.20	3.18 ± 3.21	3.19 ± 3.14	0.00(1.000)
Numbness of hands and feet	3.14 ± 3.11	2.73 ± 2.95	2.84 ± 2.99	4.11 ± 3.31	6.55(.002)^a,b^^<^^c^
Appetite	2.92 ± 2.83	3.16 ± 2.94	2.78 ± 2.66	2.73 ± 2.85	0.86(.422)
Constipation	2.59 ± 2.78	2.73 ± 3.09	2.45 ± 2.65	2.54 ± 2.42	0.35(.707)
Difficulty swallowing	1.73 ± 2.42	1.69 ± 2.41	1.49 ± 2.24	2.06 ± 2.63	1.47(.233)
Diarrhea	1.66 ± 2.37	1.40 ± 2.31	1.77 ± 2.19	1.92 ± 2.62	1.55(.213)

^a,^^b,c^Tukey HSD test

*SD* standard deviation

Among the symptoms, the fatigue score was highest at 5.56 ± 2.69 points, and did not differ significantly by survival stage. Fatigue had the highest score in all stages of survival and was the second highest symptom score for skin/hair changes in the acute stage, cold/hot intolerance in the extended stage, and sleep in the permanent stage (Table 3).

### Performance of health-promoting behavior by subject characteristics, social support, self-efficacy, symptoms, and fear of recurrence

The overall score for engaging in health-promoting behavior was significantly higher in subjects who had more than two radioactive iodine treatments than in those who had one (Table 4).

**Table 4:** Health-promoting behavior according to the characteristics of thyroid cancer survivors (*N* = 354)

Characteristics	Categories	Health-promoting behavior	
		Mean ± SD	*F*/t (*p*)
Age (years)	< 40	100.97 ± 13.01	
	40–49	101.48 ± 14.59	2.52(.058)
	50–59	98.82 ± 16.89	
	≥ 60	107.55 ± 17.56	
Gender	Men	100.29 ± 16.07	− 0.4(.692)
	Women	101.41 ± 15.01	
Marital status	Married	101.33 ± 15.05	0.03(.978)
	Single	101.27 ± 15.37	
Education	≤ High school	102.14 ± 15.98	0.55(.585)
	≥ College	101.08 ± 14.85	
Monthly income	< 200	101.21 ± 15.97	
(10,000 Korean won)^1^	200 to < 400	98.47 ± 14.04	1.96(.120)
	40 to < 600	101.35 ± 14.91	
	≥ 600	103.42 ± 15.59	
Employment status	Employed	101.12 ± 15.16	− 0.29(.771)
	Not employed	101.59 ± 15.03	
Frequency of accessing online community	Every day	101.92 ± 14.87	
	2–3 times a week	100.64 ± 16.92	0.73(.533)
	2–3 times a month	99.19 ± 14.53	
	Rarely accessed	103.62 ± 11.59	
Cancer type	Papillary	101.18 ± 15.11	− 0.78(.437)
	Other^1^	104.19 ± 14.64	
Extent of surgery	Less-than-total^2^	101.43 ± 14.78	0.41(.665)
	Total thyroidectomy	100.34 ± 14.98	
	Other^3^	102.24 ± 15.86	
Cancer recurrence	Yes	105.07 ± 13.94	1.37(.170)
	No	100.99 ± 15.16	
Number of I^131^ therapies^4^	None^a^	101.76 ± 14.66	3.29(.039) b<c
	1 time^b^	98.78 ± 15.21	
	≥ 2 times^c^	104.77 ± 15.53	

*SD* standard deviation

^1^Other includes follicular cancer, Hurthle cell cancer, medullary cancer, poorly differentiated cancer, and anaplastic cancer

^2^Less-than-total includes less-than-total thyroidectomy and hemithyroidectomy

^3^Other includes total thyroidectomy along with lateral neck dissection and re-operation

^4^I^131^ therapies mean radioactive iodine therapies

^a,b,c^Tukey HSD test

The performance in health-promoting behaviors among patients with thyroid cancer correlated significantly with social support, self-efficacy, symptoms, and fear of recurrence (Table 5).

**Table 5:** Correlations among health-promoting behavior, social support, self-efficacy, symptoms, and fear of recurrence (*N* = 354)

Variables	Health-promoting behavior	Social support	Self-efficacy	Symptoms	Fear of recurrence
	*r* (*p*)	*r* (*p*)	*r* (*p*)	*r* (*p*)	*r* (*p*)
Health-promoting behavior	1				
Social support	.46 (< .001)	1			
Self-efficacy	.51 (< .001)	.47 (< .001)	1		
Symptoms	− .29 (< .001)	− .23 (< .001)	− .34 (< .001)	1	
Fear of recurrence	− .13 (0.016)	− .07 (0.186)	− .40 (< .001)	.25 (< .001)	1

### Factors of health-promoting behavior by survival stage

The factors affecting health-promoting behavior in acute survival were self-efficacy and social support. In the extended stage, symptoms, social support, fear of recurrence, and the number of radioactive iodine treatments significantly affected health-promoting behavior. In the permanent stage, self-efficacy, social support, the number of radioactive iodine treatments, and age significantly affected health-promoting behavior (Table 6).

**Table 6:** Factors of health-promoting behavior by survival stage (*N* = 354)

Variables	Categories	Acute stage (*n* = 147), *β* (*p*)	Extended stage (*n* = 112), *β* (*p*)	Permanent stage (*n* = 95), *β* (*p*)
Social support		.30 (.001)	.25 (.011)	.26 (.007)
Self-efficacy		.43 (< .001)	.17 (.124)	.42 (< .001)
Fear of recurrence		.03 (.676)	.21 (.032)	.09 (.358)
Symptoms		− .03 (.662)	− .34 (< .001)	− .11 (.219)
Number of I^131^ therapies^1^ (ref: ≥ 2 times)	None	− .15 (.391)	− .21 (.067)	− .06 (.535)
	1 time	− .16 (.337)	− .25 (.032)	− .29 (.002)
Age (years) (ref: ≥ 60)	< 40	− .09 (.561)	− .32 (.102)	− .14 (.151)
	40–49	− .08 (.636)	− .36 (.087)	− .30 (.007)
	50–59	− .06 (.687)	− .15 (.357)	− .24 (.018)
Monthly income (10,000 won)^2^ (ref: ≥ 600)	< 200	.07 (.349)	.01 (.954)	− .04 (.648)
	200– < 400	.01 (.874)	.15 (.183)	− .08 (.407)
	400– < 600	.05 (.530)	.02 (.857)	− .01 (.925)
Employment status (ref: Not employed)	Employed	− .04 (.585)	.14 (.117)	.14 (.082)

^1^I^131^ therapies mean radioactive iodine therapies

^2^Incomes are shown in 10,000 South Korean won (KRW) units

## Discussion

### Health-promoting behaviors of thyroid cancer survivors

The average score for health-promoting behaviors among patients with thyroid cancer was 2.67/4.00. Among the sub-areas of health-promoting behaviors reported by thyroid cancer survivors in this study, exercise was the lowest with 2.36 points, and stress management the second lowest with 2.46 points. This was similar to the findings among patients with breast cancer, whose scores were lowest for stress management and second lowest for exercise [33]. In addition, it has been found that most cancer patients are not sufficiently active [34]. Exercise is known to improve the quality of life and fitness of cancer survivors; an effective exercise strategy is important [35].

Of the thyroid cancer patients, 53.1% are in their 40 s and 50 s. Thyroid cancer is three times more common in women than in men [3]. In addition, women in their 40 s and 50 s who have survived thyroid cancer need to take steps to prevent osteoporosis, in preparation for menopause because taking thyroid hormones raises the risk of osteoporosis [36]. Therefore, it is necessary to develop an exercise program for women in their 40 s and 50 s to prevent osteoporosis.

### Characteristics of thyroid cancer survivors by survival stage

Analysis of the survival stage characteristics of thyroid cancer survivors revealed that many patients did not receive radioactive iodine therapy and did not experience recurrence. In the permanent stage, the proportion of patients who received radioactive iodine treatment more than 2 times and experienced recurrence was high. Radioiodine treatment is performed after surgery, as needed, and the next radioiodine dose can be administered after 6 to 12 months [23]. Survivors of thyroid cancer underwent surgery in the acute stage and were followed up and given radioactive iodine treatment. Recurrence could have been experienced.

Social support was low in the permanent stage, which is consistent with the existing research results that social support weakened after the course of treatment [37, 38]. Thyroid cancer survivors experience psychological distress and side effects, but in general, thyroid cancer is known to have a high survival rate and a good prognosis, which can make it difficult to get help [39, 40]. In this study, it was found that meaningful support from others was significantly lowered during the permanent stage, and it was necessary to provide adequate social support to patients at all stages.

Fear of recurrence is a common problem experienced by patients with thyroid cancer, and previous studies have also shown that patients with thyroid cancer have a high fear of recurrence [41, 42]. In this study, the fear of recurrence score of thyroid cancer survivors was highest in the acute stage. Acute stage patients are thought to have a great fear of recurrence because they are diagnosed with cancer and undergo surgery. As information about health threats can positively contribute to fear control, it would be helpful to provide enough information about thyroid cancer in the acute stage [43].

In this study, the sum of symptoms did not differ significantly according to survival stage. Fatigue was the highest in the sub-domain score of symptoms, and many studies have reported that the fatigue score of thyroid cancer patients is very high and long-lasting [44, 45]. Sleeping difficulties were found in the permanent stage, and a study of breast cancer patients found that quality-of-life symptoms improved over time, but sleep problems persisted until after 5 years [46]. In addition, a study of ovarian cancer patients showed that symptoms appeared differently depending on the stage of survival [47]. As experiences change, it may be helpful to provide interventions to predict the next stage of symptoms.

### Factors influencing health-promoting behavior by survival stage

The factors affecting the use of health-promoting behavior among thyroid cancer survivors differed by survival stage. Fear of recurrence and symptoms influenced health-promoting behavior only in the extended stage, and age was associated with health-promoting behavior only in the permanent stage. Social support was found to be a variable influencing health-promoting behavior in all stages of survival, and social support was low in the permanent stage.

The acute stage is a period of active treatment, and it is necessary to promote self-efficacy and encourage health-promoting behavior through social support. A diagnosis of cancer can be a motivation to change health behaviors [19]. Motivation is important in changing health behaviors; therefore, it is important to strengthen motivation in the early stages [48, 49]. In the acute stage, medical staff should learn about each patient’s health behaviors and plan for change together with them.

As survivors at the extended survival return to work after completing treatment, they engage in relatively fewer health-promoting behaviors; hence, active education is needed to strengthen their commitment to health-promoting behavior [20]. As shown by this study, in the extended stage, it is necessary to focus on managing the symptoms and fear of recurrence. In this study, high symptom scores correlated with low health-promoting behavior scores in a dose-dependent manner. Previous studies have also found that physical symptoms experience reduced health-promoting behavior [14]. A study of health-promoting behavior among middle-aged women found that more severe menopausal symptoms correlated with low health-promoting behavior [50]. Patients with thyroid cancer need continuous intervention for their symptoms because fatigue persists for a long time after treatment [51]. The results of this study also show that in the extended stage, symptoms interfere with health promotion. Nurses should thus help patients acquire skills to cope with symptoms, such as fatigue, which interfere with health promotion and provide positive feedback to encourage them to continue their health behaviors.

Among thyroid cancer survivors in the extended stage, a high fear of recurrence correlated with a high use of health-promoting behaviors. This is a principle of the health belief model, which is based on an awareness of the risks of disease and the value of preventive health behavior [13]. However, fear of recurrence needs to be approached carefully because a high fear of recurrence carries a high level of patient anxiety [30]. Previous studies have shown that providing information about benefits effectively motivates health behaviors more than focusing on potential losses [52]. Therefore, the fear of recurrence needs to lead to effective health behavior by emphasizing the benefits of health behavior and providing accurate information. Fear of recurrence is also related to unmet needs. It is necessary to develop programs that meet their needs [53].

Patients with thyroid cancer who received radioactive iodine treatment more than once reported high health-promoting behavior scores in this study, which is consistent with higher health-promoting behavior scores in patients with breast cancer who received chemotherapy [33]. In a previous study, the severity-of-disease awareness in patients with thyroid cancer correlated with the number of radioactive iodine treatments, and patients who received radioactive iodine treatment more than once were thought to have attempted to improve their health by recognizing the severity of their disease [54].

A program for continuous health promotion should be developed for patients in the permanent stage of survival, with interventions offered to increase self-efficacy and social support. Stages of thyroid cancer are divided according to age, and at diagnosis, patients over 55 years of age have a higher cancer stage and a worse prognosis [55]. Among respondents in the permanent stage, health-promoting behavior was higher in survivors older than 60 years, possibly because they perceived the risk of thyroid cancer. Therefore, education is needed to encourage health-promoting behavior among survivors in their 40 s and 50 s. The number of online users in their 40 s and 50 s is high. In this study, 54% of respondents said that they accessed the online community every day [56]. In recent years, e-health applications and healthcare education using mobile phones have been highlighted and reported to have positive effects [57, 58]. In the permanent stage of survival, the interval between outpatient visits is long, so it would be helpful to develop an online healthcare program to provide disease-related information and encourage regular exercise.

This study has confirmed that self-efficacy and social support are factors that influence health promotion, as shown in Pender’s health promotion model, and that the fear of recurrence and symptoms also influence cancer survivors’ health-promoting behaviors. With the development of medical technology and improvements in the long-term cancer survival rate, cancer is becoming a disease that requires long-term management. Interventions that vary with the stage of survival will help improve health-promoting behaviors among cancer survivors.

### Limitations

Most data collected in this study came from patients who responded through the online community, and it is difficult to rule out the possibility that people who frequently accessed the online community search for health information and made more efforts than those who do not. In addition, since the study was conducted in a hospital located in the city, and an online community, it is difficult to rule out a selection bias that people with high educational levels and incomes were the major participants. In addition, this study compared groups of thyroid cancer survivors in the acute, extended, and permanent survival stages, and the characteristics of these groups might have differed in meaningful ways. Because this study was cross-sectional, it did not consider the passage of time. Therefore, interpretations should be made with care when comparing the three groups. In the future, longitudinal cohort studies are needed to identify changes in health-promoting behaviors.

## Conclusion

Factors influencing the health-promoting behaviors of thyroid cancer survivors differed at each survival stage. Therefore, interventions to promote appropriate health behaviors should be developed, to reflect the characteristics of each survival stage. It is necessary to strengthen the management of survivors not only at the acute stage, but also in the extended and permanent stages.

## Data Availability

The authors confirmed that some access restrictions apply to the data underlying the findings. The data from this research study cannot be shared publicly due to concerns of participant confidentiality. If there is a researcher who wants to receive the data of this study, contact the corresponding author (sangheekim@yuhs.ac). Data are available from the Gangnam Severance Hospital Ethics Committee for researchers who meet the criteria for access to confidential data.
